# $hh+\text {Jet}$ production at 100 TeV

**DOI:** 10.1140/epjc/s10052-018-5788-y

**Published:** 2018-04-21

**Authors:** Shankha Banerjee, Christoph Englert, Michelangelo L. Mangano, Michele Selvaggi, Michael Spannowsky

**Affiliations:** 1 Institute for Particle Physics Phenomenology, Department of Physics, Durham University, Durham, DH1 3LE UK; 2 Université Grenoble Alpes, USMB, CNRS, LAPTh, 74000 Annecy, France; 3 School of Physics and Astronomy, University of Glasgow, Glasgow, G12 8QQ UK; 4 CERN, 1211 Geneva, Switzerland

## Abstract

Higgs pair production is a crucial phenomenological process in deciphering the nature of the TeV scale and the mechanism underlying electroweak symmetry breaking. At the Large Hadron Collider, this process is statistically limited. Pushing the energy frontier beyond the LHC’s reach will create new opportunities to exploit the rich phenomenology at higher centre-of-mass energies and luminosities. In this work, we perform a comparative analysis of the $hh+\text {jet}$ channel at a future 100 TeV hadron collider. We focus on the $hh\rightarrow b\bar{b} b\bar{b}$ and $hh \rightarrow b\bar{b} \tau ^+\tau ^-$ channels and employ a range of analysis techniques to estimate the sensitivity potential that can be gained by including this jet-associated Higgs pair production to the list of sensitive collider processes in such an environment. In particular, we observe that $hh \rightarrow b\bar{b} \tau ^+\tau ^-$ in the boosted regime exhibits a large sensitivity to the Higgs boson self-coupling and the Higgs self-coupling could be constrained at the 8% level in this channel alone.

## Introduction

The observed lack of any conclusive evidence for new interactions beyond the Standard Model (BSM) during the LHC’s run-1 and the first 13 TeV analyses has tightly constrained a range of well-motivated BSM scenarios. For instance, the ATLAS and CMS collaborations have already set tight limits on top partners in supersymmetric (e.g. [1, 2]) and strongly-interacting theories (e.g. [3, 4]), which makes a natural interpretation of the TeV scale after the Higgs boson discovery more challenging than ever.

With traditional BSM paradigms facing increasing challenges as more data becomes available, a more bottom-up approach to parametrising potential new physics interactions has received attention recently. By interpreting Higgs analyses using Effective Field Theory (EFT), any heavy new physics scenario that is relevant for the Higgs sector can be investigated largely model-independently [5, 6], at the price of many ad hoc interactions to lowest order [7] in the EFT expansion.

Current measurements as well as first extrapolations of these approaches to the high luminosity (HL) phase of the LHC have provided first results as well as extrapolations of EFT parameters [8–12]. One of the parameters, which is particularly sensitive to electroweak symmetry breaking potential yet with poor LHC sensitivity prospects is the Higgs self-interaction. Constraining the trilinear self-interaction directly requires a measurement of (at least) $pp \rightarrow hh$ [13–17]; accessing quartic interactions in triple Higgs production is not possible at the LHC [18, 19] and seems challenging at future hadron colliders at best [20, 21]. Early studies of the LHC’s potential to observe Higgs pair production have shown the most promising channels to be the $hh\rightarrow b\bar{b} \gamma \gamma$ [22] and $hh\rightarrow b\bar{b} \tau ^+\tau ^-$ channels [23, 24]. Recent projections by ATLAS [25] and CMS [26], based on an integrated luminosity of 3 ab$^{-1}$ and on the pileup conditions foreseen for the HL-LHC, estimate a sensitivity to the di-Higgs signal in the range of 1–2$\sigma$. Recent phenomenological papers [27–29], combining the sensitivity to several different di-Higgs final states, reach similar conclusions. ATLAS [25] quotes a sensitivity to the value of the Higgs self-coupling (assuming SM-like coupling values for all other relevant interactions) in the range of $-0.8< \lambda /\lambda _{SM} < 7.7$, at 95% confidence limit. Improving this sensitivity baseline is one of the main motivations of future high energy hadron colliders, and proof-of-principle analyses suggest that a vastly improved extraction of trilinear Higgs coupling should become possible [30–34] at a future 100 TeV collider.

Most of these extrapolations have focused on gluon fusion production $p(g)p(g)\rightarrow hh$. Owing to large gluon densities at low momentum fractions, the associated di-Higgs cross section increases by a factor of $\sim 39$ compared to 14 TeV collisions [35, 36], with QCD corrections still dominated by additional unsuppressed initial state radiation [37–43]. While the process’ kinematic characteristics of Higgs pair production remain qualitatively identical to the LHC environment, extra jet emission becomes significantly less suppressed leading to a cross section enhancement of $pp\rightarrow hhj$ of $\sim 80$^1^ compared to 14 TeV collisions. This provides another opportunity for the 100 TeV collider: Since the measurement of the self-coupling is largely an effect driven by the top quark threshold [17], accessing relatively low di-Higgs invariant masses is the driving force behind the self-coupling measurement. In fact, recoiling a collimated Higgs pair against a jet kinematically decorrelates $p_{T,h}$ and $m_{hh}$. Compared to $pp\rightarrow hh$, it thus exhibits a much higher sensitivity to the variation of the Higgs trilinear interaction while keeping $p_{T,h}$ large [24], which is beneficial for the reconstruction and separation from backgrounds. However, such an approach is statistically limited at the LHC. Given the large increase in $pp\rightarrow hh+\text {jet}$ production in this kinematical regime as well as the increased luminosity expectations at a 100 TeV collider, it can be expected that jet-associated Higgs pair production can add significant sensitivity to self-coupling studies at a 100 TeV machine.

Quantifying this sensitivity gain in a range of exclusive final states with different phenomenological techniques is the purpose of this work. More specifically we consider final states with largest accessible branching fractions $hh \rightarrow b\bar{b} b\bar{b}$ [23, 44, 45] and $hh \rightarrow b\bar{b} \tau ^+ \tau ^-$ [23, 24, 46], where we also differentiate between leptonic and hadronic $\tau$ decays (and consider their combination).

This work is organised as follows: We consider the $b\bar{b} \tau \tau$ channel in Sect. 2. In particular we compare the performance gain of a fully-resolved di-Higgs final state analysis extended by substructure techniques highlighting the importance of high-transverse momentum Higgs pairs that are copious at 100 TeV. We discuss the $b\bar{b} b\bar{b}$ channel in Sect. 3.

## The $jbb\tau \tau$ channels

### General comments

Let us first turn to the $jbb\tau \tau$ channels. We will see that these are more sensitive to variations of the trilinear Higgs coupling and they therefore constitute the main result of this work. This is in line with similar studies at the LHC (see Refs. [24, 44, 46]) that show that the signal vs. background ratio can be expected to be better for this channel than for the four *b* case.

We study the various decay modes of the taus and consider two exclusive final states, purely leptonic tau decays $h\rightarrow \tau _{\ell } \tau _{\ell }$ and mixed hadronic-leptonic decays $h\rightarrow \tau _{\ell } \tau _h$, where the subscripts $\ell$ and *h* denote the leptonic (to $e, \mu$) and hadronic decays of the taus, respectively. The scenario involving the purely hadronic decays, $h\rightarrow \tau _h \tau _h$ will undoubtedly add to the significance. However, scenarios involving two hadronic taus will incur stronger QCD backgrounds and hence we will need to simulate various fake backgrounds and will also require an accurate knowledge of the $j \rightarrow \tau _j$ fake rate, where *j* denotes a light jet. At this stage, we do not feel confident that we can reliably estimate these fake backgrounds, and hence neglect this decay mode in the present study.

There are three categories of backgrounds that we consider for this scenario. The most dominant background results from $t\bar{t}j$ with the leptonic top decays ($t \rightarrow b W \rightarrow b \ell \nu$), which includes decays to all the three charged leptons.^2^ Furthermore, we have the pure EW background and a mixed QCD-EW background of $j b \bar{b} \tau ^+ \tau ^-$.^3^ The pure EW and QCD+EW processes consist of various sub-processes. A typical example for the pure EW scenario is $pp\rightarrow HZ/\gamma ^* + \; \text {jet} \rightarrow b\bar{b}\tau ^+\tau ^- + \; \text {jet}$. Whereas, for the QCD+EW processes, a typical example is $pp\rightarrow b\bar{b} Z/\gamma ^* + \; \text {jet} \rightarrow b\bar{b}\tau ^+\tau ^- + \; \text {jet}$. In all these background processes, either from the $\tau$ decays or from the *W*-boson decays (for the $t\bar{t}j$ background), we may encounter leptons ($e,\mu$). There are potentially other irreducible backgrounds like $W \; (\rightarrow \ell \nu )+$ jets but these turn out to be completely subdominant when compared to the other backgrounds. This is shown in the context of the $hh \rightarrow bb\tau \tau$ present and future analyses by ATLAS [47] and CMS [48, 49]. Similar conclusions will hold in the present study. Hence we neglect such backgrounds from our present analysis. All samples, including the signal, are generated with MadGraph5_aMC@NLO [50] in Born-level mode, and we neglect effects from jet merging up to higher jet multiplicities. For our signal samples, the Higgs bosons are decayed using MadSpin [51, 52]; the showering is performed using Pythia 8 [53]. To account for QCD corrections we use global *K* factors for the signal of $K=1.8$ for the EW contributions (extrapolating from [54]), $K=1.5$ for the QCD+EW contribution [55] as well as $K=1.0$ for $t\bar{t} j$ following [56].

To operate with an efficient Monte Carlo tool chain, we generate the EW and mixed QCD+EW events with the following generator level cuts: $p_T^{b} > 23$ GeV, $p_T^{\ell }> 8$ GeV, $|\eta ^{b,\ell }| < 3$, $p_T^j > 100$ GeV, $|\eta ^j| < 5$, $\Delta R_{b,b}>0.2$, $\Delta R_{\ell \ell } > 0.15$, $\Delta R_{b/j,\ell }>0.3$, $90~\text {GeV}< M_{b,b} < 160~\text {GeV}$ and $90~\text {GeV}< M_{\ell , \ell } < 200~\text {GeV}$, where $\ell = e, \mu , \tau$ and *b* denotes final state bottom quarks. *R* is the azimuthal angle—pseudo-rapidity ($\phi$–$\eta$) distance and *M* denotes invariant masses. The same requirements are imposed on $t\bar{t} j$, however, without a lower bound on $M_{\ell \ell }$. The only event generator cut applied to the signal is transverse momentum cut on the light flavor jet $p_T^j > 100$ GeV.

Given the discriminating power of $m_{T2}$ which was motivated in Ref. [46] to reduce the $t\bar{t}$ background, we consider a similar variable with the aim to reduce the dominant $t\bar{t}+{\text {jet}}$ background. The top background final state can be described schematically through a decay chain

2.1a $$\begin{aligned} A\rightarrow & {} B + C\end{aligned}$$

2.1b $$\begin{aligned} A\rightarrow & {} B' + C', \end{aligned}$$

where $B,B'$ ($C,C'$) denote the visible (invisible) decay products of the top branching ($A=t,\bar{t}$). For such a branching one can construct the $m_{T2}$ variable [57]

2.1c $$\begin{aligned}&m_{T2}(m_B,m_{B'},\mathbf {b}_T,\mathbf {b}'_T,{\mathbf {p}}_T^{\Sigma },m_C,m_{C'})\nonumber \\&\quad \equiv \underset{\mathbf {c}_T + \mathbf {c}'_T ={\mathbf {p}}_T^{\Sigma }}{\text {min}} {\text {max}(m_T,{m}'_T)}, \end{aligned}$$

where $m_T$ denotes the transverse mass constructed from $\mathbf {b}_T$, $\mathbf {c}_T$ and $m_B$

2.1d $$\begin{aligned} m_T^2(\mathbf {b}_T, \mathbf {c}_T, m_B, m_C) \equiv m_B^2 + m_C^2 + 2(e_B e_C - \mathbf {b}_T \cdot \mathbf {c}_T), \end{aligned}$$

with transverse energy $e_i^2 = m_i^2 + {\mathbf {p}}_{i,T}^2$, $i=B,C$. $m'_T$ refers to the same observable calculated from the primed quantities in Eq. (2.1). The minimisation in Eq. (2.1c) is performed over all momenta $\mathbf {c}_T$ and $\mathbf {c}'_T$, subject to the condition that their sum needs to reproduce the correct ${\mathbf {p}}_T^{\Sigma }$, which is normally chosen to coincide with the overall missing energy . However, because the tau’s decay is partially observable, we can modify the $m_{T2}$ definition to include the visible transverse momenta of the tau leptons by identifying2.1e As we will see below, this modified $m_{T2}$ plays a crucial role in suppressing the dominant $t\bar{t}j$ background. We must emphasise here that many distinctly different definitions of $m_{T2}$ have been considered in Ref. [58]. The authors in Ref. [46] have considered several such definitions of the $m_{T2}$ variable and found them having very similar discriminatory power.

### The resolved $\tau _{\ell } \tau _{\ell }$ channel

The leptonic di-tau final states are undoubtedly the cleanest channels out of the three di-tau options. We can identify exactly two leptons ($e, \mu$), two *b*-tagged jets and at least one hard non *b*-tagged jet. We therefore pre-select the events by requiring the following cuts at reconstruction level:^4^ jets are clustered with size 0.4 and $p_T^{j}>30$ GeV in $|\eta |<4.5$; the hardest jet is required to have $p_T^{j_1}>105~\text {GeV}$. Leptons are required to have $p_T^{\ell }>10~\text {GeV}$ and $|\eta | < 2.5$. We require two leptons and select two jets with $p_T > 30$ GeV and $|\eta | < 2.5$, which are subsequently *b*-tagged. All objects need to be well separated $\Delta R(b,b/j_1/\ell ),\Delta R(\ell ,j_1) >0.4$ and $\Delta R(\ell ,\ell )>0.2$. To efficiently suppress the *Z*-induced background we demand $105~\text {GeV}< M_{b,b} < 145 ~\text {GeV}$. Furthermore we require a significant amount of missing energy  GeV.

After these pre-selection requirements we apply a boosted decision tree (BDT) analysis which is the experiments’ weapon of choice when facing a small signal vs. background ratio (see e.g. the very recent ATLAS $t\bar{t} h$ analysis [62]). We include a large amount of (redundant) kinematic information^5^ to the training phase, as listed in Table 1.^6^

We focus on a training of the boosted decision tree for a SM-like value of the trilinear Higgs coupling $\lambda _{\text {SM}}$. We employ the boosted decision tree algorithm of the TMVA framework [63] on the basis of 30 $\text {ab}^{-1}$ of data at 100 TeV. Our results are tabulated in Table 2. As can be seen, we can typically expect small signal vs background ratios at small signal cross sections. The latter is mostly due to the small fully-leptonic branching ratios of the tau pairs.

### The resolved $\tau _{\ell } \tau _{h}$ channel

Given the small *S* / *B* for the fully leptonic channel of the previous section we consider the case where one tau lepton decays leptonically while the other tau decays hadronically.

Recently, a major CMS level-1 trigger update has increased the hadronic tau tagging efficiency by a factor of two [65–67] for tau candidates with $p_T\gtrsim 20~\text {GeV}$, robust against pile-up effects. Fully-hadronic di-tau decays of the Higgs boson for 13 TeV collisions can be tagged at 70% with a background rejection of around 0.999. These improvements suggest that a single tau tagging performance of 70% in a busier environment of the *hhj* final state at 100 TeV is not unrealistic and we adopt this working point in the following, assuming a sufficiently large background rejection for fakes to be negligible.

**Fig. 1:**
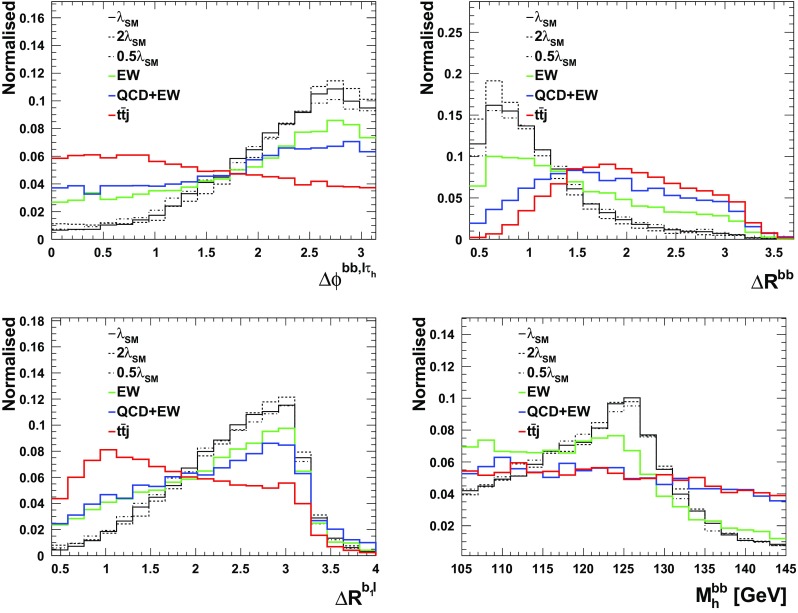
Normalised differential distributions that serve to isolate signal from background in the $\tau _{\ell } \tau _h$ case in the BDT analysis

**Fig. 2:**
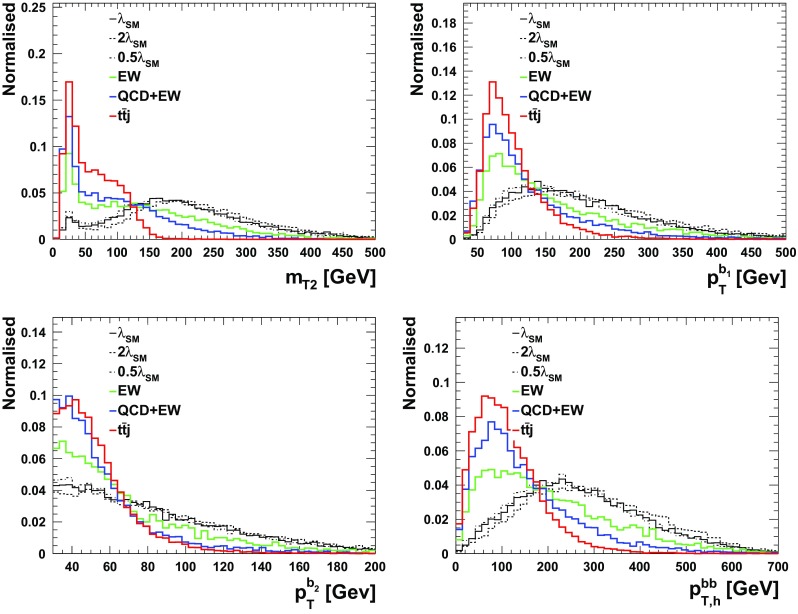
Normalised differential distributions that serve to isolate signal from background in the $\tau _{\ell } \tau _h$ case in the BDT analysis

**Fig. 3:**
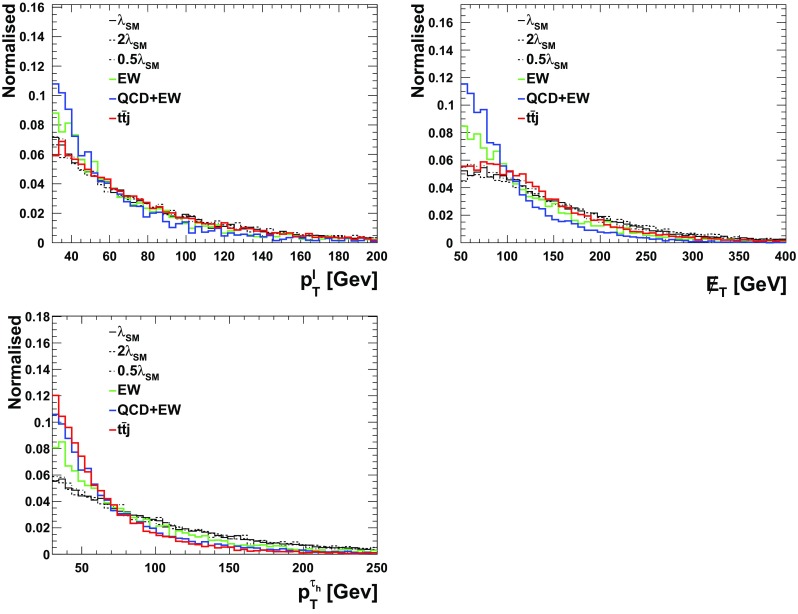
Normalised differential transverse momentum contributing to the $\tau _{\ell } \tau _h$ BDT analysis

We follow the analysis of the previous section and employ similar variables for the BDT. The only difference here is that here we demand 2 *b*-tagged jets, one $\tau$-tagged jet, one lepton and at least one hard non $b,\tau$-tagged jet. All the aforementioned variables for the $\tau _{\ell } \tau _{\ell }$ scenario, Table 1, can be utilised here with the only difference of replacing one lepton by a $\tau _h$.^7^ The distributions are shown in Figs. 1, 2 and 3 and the results are tabulated in Table 3. As can be seen, different to the fully-leptonic case, the increase in signal allows us to suppress the dominant $t\bar{t}j$ background further without compromising the signal count too much. This leads to a much larger expected sensitivity in the $\tau _\ell \tau _h$ channels.

**Table 1:** Observables included in the boosted decision tree for the leptonic $\tau$ channels of the $pp\rightarrow hhj$ analysis of Sect. 2.2

Observable	Reconstructed object
$p_T$	2 *b*-tagged jets
	2 leptons
	Hardest non *b*-tagged jet
	*bb* system
	$\ell \ell$ system
$p_T$ ratios	2 *b*-tagged jets
	2 leptons
$\Delta R$	2 *b*-tagged jets
	2 leptons
	*b*-tagged jets and jet $j_1$/leptons
	Leptons and jet $j_1$
*M*	2 *b*-tagged jets
	2 leptons
	*b*-tagged jets and leptons
$m_{T2}$	Described in Eq. (2.1)
$\Delta \phi$	Between *bb* and $\ell \ell$ systems
	Reduce sub-leading backgrounds

**Table 2:** Results of the fully leptonic tau decay channels outlined in Sect. 2.2 in femtobarns (numbers to the left of the double vertical lines) after an optimised cut on the BDT output. We include results for three different choices of the self-coupling within the $\kappa$ framework [64], $\kappa _\lambda =\lambda /\lambda _{\text {SM}}$; BDT training is performed with $\lambda =\lambda _{\text {SM}}$

	Signal	QCD+EW	EW	$t\bar{t} j$	Tot. background	*S* / *B*	$S/\sqrt{B}$, 30/ab
$\kappa _\lambda =1/2$	0.070	0.26	0.04	1.25	1.55	0.046	9.85
$\kappa _\lambda =1$	0.059					0.038	8.19
$\kappa _\lambda =2$	0.043					0.028	5.98

Combining the results of the previous section with the $\tau _\ell \tau _h$ results into a log-likelihood CLs hypothesis test [68–70] assuming the SM as null hypothesis values of (assuming no systematic uncertainties)

2.2 $$\begin{aligned} 0.65< & {} \kappa _\lambda < 1.44 \quad 3/\text {ab}, \end{aligned}$$

2.3 $$\begin{aligned} 0.88< & {} \kappa _\lambda < 1.13 \quad 30/\text {ab}, \end{aligned}$$

at 68% confidence level. Here, $\kappa _{\lambda } = \lambda /\lambda _{\text {SM}}$, is the measure of the deviation of the Higgs trilinear coupling with respect to the SM expectation.

### The significance of high-$p_T$ final states

So far our strategy has focused on resolved particle-level objects without making concessions for the larger expected sensitivity of the high $p_T$ final states. Jet-substructure techniques (see e.g. [71]) are expected to be particularly suited for kinematic configurations for which $h\rightarrow b\bar{b}$ recoils against the light-flavor and hard jet [24], while the $h\rightarrow \tau \tau$ decay happens at reasonably low transverse momentum. This way, although one Higgs is hard, low invariant Higgs pair-masses can be accessed from an isotropic $h\rightarrow \tau \tau$ decay given a collimated $b\bar{b}$ pair. This particular kinematic configuration is not highlighted in the previous section and we can expect that the sensitivity of Eq. (2.22.3) will increase once we focus with jet-substructure variables on this phase-space region which is highly relevant for our purposes. The benefit of this analysis will hence be two-fold: firstly we will exploit the background rejection of the non-Higgs final states through the adapted strategies of jet-substructure techniques. And secondly we will directly focus on a phase space region where we can expect the impact of $\kappa _\lambda \ne 1$ to be most pronounced.

To isolate this particular region, we change the analysis approach of Sects. 2.2 and 2.3. Before passing the events to the BDT we require at least two so-called fat jets of size $R=1.5$ and $p_T^{j} > 110~\text {GeV}$. One of these fat jets is required to contain displaced vertices associated with B mesons. We remove the jet constituents (that can contain leptons) and re-cluster the event along with our standard anti-kT choice. We then require either two isolated leptons ($\tau _\ell \tau _\ell$ cases) or one isolated lepton together with one $\tau$-tagged jet ($p_T>30~\text {GeV}$) using again a tagging efficiency of 70% ($\tau _\ell \tau _h$ cases). All these objects are required to be in the central part of the detector $|\eta | <2.5$. Subsequently we apply substructure techniques to the jet containing displaced vertices following the by-now standard procedure of Ref. [71] (we refer the reader for details to this publication and limit ourselves to quoting our choices of mass drop parameter 0.667 and $\sqrt{y}=0.3$). After jet-filtering we double-*b* tag the two hardest subjets with an efficiency of 70% (2% mistag rate) and require the identified B-mesons to have $p_T>25~\text {GeV}$.^8^ Finally, we require the leptons to be separated by $\Delta R(\ell \ell )>0.2$ in the $\tau _\ell \tau _\ell$ case. In the $\tau _h\tau _\ell$ case we require the lepton to be sufficiently well-separated from the hadronic tau $\Delta R(\ell ,\tau _h)>0.4$.

**Fig. 4:**
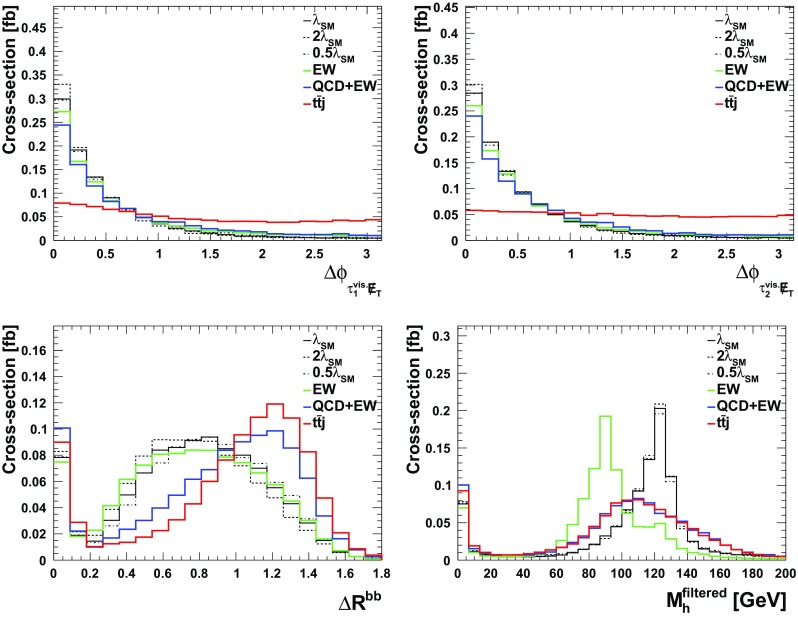
Discriminating observables contributing to the boosted analysis of Sect. 2.4

**Fig. 5:**
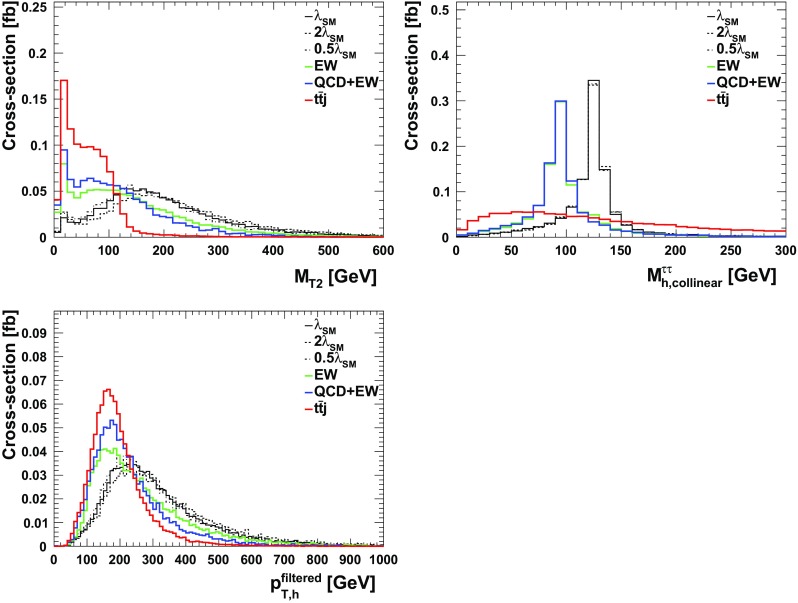
Discriminating observables contributing to the boosted analysis of Sect. 2.4

We use the (jet-substructure) observables of Table 4 as BDT input^9^ (for a discussion of redundancies of the used observables see below). The signal vs. background discriminating power is shown in Figs. 4 and 5. We can increase the sensitivity of the signal by using the collinear approximation outlined in Ref. [72] for the $\tau \tau$ pair.

The combined results are tabulated in Table 5. As can be seen, this approach retains larger signal and background cross sections compared to the fully-resolved approach that has a combined $S/B\simeq 0.08$. The sensitivity to $\kappa _\lambda$ is slightly more pronounced in the jet-substructure approach as expected. Together with the increased statistical control we can therefore constrain $\kappa _\lambda$ slightly more tightly (assuming again no systematic uncertainties)

2.4 $$\begin{aligned}&0.76< \kappa _\lambda < 1.28 \quad 3/\text {ab}, \end{aligned}$$

2.5 $$\begin{aligned}&0.92< \kappa _\lambda < 1.08 \quad 30/\text {ab}, \end{aligned}$$

at 68% confidence level using the identical CLs approach as above.

Before concluding this section we note that for our $b\bar{b} \tau ^+ \tau ^-$ analyses, the *S* / *B* values are 10% or more for the boosted combined ($\tau _{\ell } \tau _h + \tau _{\ell } \tau _{\ell }$) analysis and the resolved $\tau _l \tau _h$ analysis. For the $\tau _{\ell } \tau _{\ell }$ analysis however, we get *S* / *B* below 5%. Such values of *S* / *B* are not uncommon in Higgs analyses at the LHC. For example the *S* / *B* in the inclusive $H\rightarrow \gamma \gamma$ search is 1/30, and in the observation of $VH(\rightarrow b\bar{b})$, the *S* / *B* is in the range of 1–2% [73], depending on the vector boson decay mode. Ultimately, what counts is the precision with which the background rate can be determined. In our case, as in the LHC examples given above, the background rate can be extracted directly from the data, using the sidebands of the various kinematical distributions that we consider.

### Comments on cut-and-count experiments and redundancies

A possible source of criticism of BDT based signal selection is that they cannot be straightforwardly mapped onto cut-and-count analyses, and the obtained signal region does not necessarily consist of connected physical phase space regions. In a busy collider environment with many competing processes and background rates that exceed the expected signal by orders of magnitude, multivariate methods are nevertheless very powerful tools that allow to extract information in various forms.^10^

**Table 3:** Results of the $h\rightarrow \tau _\ell \tau _h$ decay channels outlined in Sect. 2.3 in femtobarns (numbers to the left of the double vertical lines) after an optimised cut on the BDT output. We include results for three different choices of the self-coupling within the $\kappa$ framework [64], $\kappa _\lambda =\lambda /\lambda _{\text {SM}}$; BDT training is performed with $\lambda =\lambda _{\text {SM}}$

	Signal	QCD+EW	EW	$t\bar{t} j$	Tot. background	*S* / *B*	$S/\sqrt{B}$, 30/ab
$\kappa _\lambda =0.5$	0.169	0.52	0.07	0.37	0.96	0.176	29.81
$\kappa _\lambda =1$	0.141					0.147	24.97
$\kappa _\lambda =2$	0.105					0.109	18.49

The kinematics of $pp\rightarrow h h j$ is fully determined by five independent parameters. This raises the question whether the observed correlations of observables might allow us to consider subsets of the observables listed above. We investigate this by systematically removing correlated observables to trace their impact on our final sensitivity; we focus on the boosted selection as it shows the largest physics potential.

When removing observables which exhibit correlations of more that 70%, we find our signal yields decreased in the percent range while the background (most notably $t\bar{t}j$) increases by $\gtrsim 15\%$. The impact on the signal, although small in size, is such that the $\kappa _\lambda$-dependence of the cross section becomes flatter. In total, focussing on observables with less than 70% correlation therefore translates into constraints on the trilinear coupling $0.89< \kappa _\lambda < 1.28$ at 30/ab, which is clearly worse than the projection of Eq. (2.42.5). Decreasing our correlation threshold to 60%, we find our sensitivity even further decreased. This, together with a uniform relative importance of the observables for the BDT output score, indicates that the comprehensive list of observables indeed provides important discriminatory power, in particular when fighting against the large $t\bar{t} j$ background.

**Table 4:** Observables included to the boosted decision tree for the jet-substructure analysis of $pp\rightarrow hhj$, Sect. 2.4

Observable	Reconstructed object
$p_T$	2 hardest filtered subjets
	2 visible $\tau$ objects ($\tau _{\ell }$ or $\tau _h$)
	Hardest non $b,\tau$-tagged jet
	Reconstructed Higgs from filtered jets
	Reconstructed Higgs from visible $\tau$ final states
$p_T$ ratios	2 hardest filtered jets
	2 visible $\tau$ final state objects
$m_{T2}$	Described in Eq. (2.1)
$\Delta R$	Two hardest filtered subjets
	Two visible $\tau$ objects ($\tau _{\ell } \tau _{\ell }$ or $\tau _{\ell } \tau _{h}$)
	*b*-tagged jets and lepton or $\tau _h$
	*b*-tagged jets and jet $j_1$
	Lepton or $\tau _h$ with jet $j_1$
$M_{\tau \tau }^{\text {col}}$	Collinear approximation of $h\rightarrow \tau \tau$ mass
$M^{\text {filt}}$	Filtered $j_1$ and $j_2$ (and $j_3$ if present)
$M^{vis.}_{hh}$	Filtered jets and leptons (or lepton and $\tau _h$)
	Reduce sub-leading backgrounds
$\Delta \phi$	Between visible $\tau$ final state objects and
	Between filtered jets system and $\ell \ell$ (or $\ell$ $\tau _h$) systems
$N_{\text {jets}}$	Number of anti-$k_T$ jets with $R=0.4$

We can test the robustness of our analysis by comparing it against a more traditional cut-and-count approach. As part of the BDT analysis we can use the BDT’s observable ranking to choose rectangular cuts in a particularly adapted way. From the cut-flow documented in Table 6, we see, that we can reproduce the BDT *S* / *B* sensitivity within a factor of two.

## The *jbbbb* channel

Finally, we consider the $b \bar{b} b \bar{b} j$ channel for completeness. In order to compete with the large pure QCD background that contributes to this process and to trigger the event we need to consider very hard jets, $p_T^{j_1}\gtrsim 300~\text {GeV}$. For a more efficient background simulation, we therefore again generate the background events already with relatively hard cuts at the generator level. We choose the jet transverse momentum $p_T^j > 250$ GeV, the $\Delta R$ separation between bottom quarks and the light jet $\Delta R_{b,j}> 0.4$, bottom quark transverse momentum threshold $p_T^b > 15$ GeV, as well as bottom rapidity range $|\eta ^b| < 3.0$. Furthermore, the jet rapidity range is restricted to $|\eta ^j| < 5.0$ and we also require the bottom quarks to be separated in distance $\Delta R_{b,b} > 0.2$ as well as invariant mass $M_{b,b} > 30$ GeV.^11^ For the signal, we only impose the generation level cut, $p_T^{j_1} > 200$ GeV. Throughout this part of the analysis, we will include a flat *b*-tagging efficiency of 70% with mistag efficiency 2%.

To account for QCD corrections we use again global *K* factors as described above. In addition to the backgrounds discussed for the $\tau$ channels, we also need to include a pure QCD background leading to four final state *b* quarks. The QCD corrections for this highly-involved final state are not available. We choose to use $K=1$. We note that this is consistent with the range of *K* factors for inclusive 4 jet production discussed in Ref. [75].

### The resolved channel

The signal vs. background ratio is small for such inclusive selections. Therefore, in order to assess the sensitivity that can be reached in principle, we will again employ a multi-variate analysis strategy. Before passing the events to the multi-variate algorithm, we pre-select events according to the $hh+\text {jet}$ signal event topology. For the resolved analysis we require 4 *b*-tagged jets and at least one hard non *b*-tagged jet with $p_T^{j} > 300$ GeV. The *b*-tagged jets are required to have a minimum $p_T$ of 30 GeV and need to fall inside the central detector region $|\eta ^b| < 2.5$. All reconstructed objects need to be separated by $\Delta R >0.4$. Furthermore we define two masses: Firstly, $M_h^{\text {min},M}$ which is the reconstructed Higgs masses from pairing *b*-tagged jets close to the Higgs mass of 125 GeV. And secondly, $M_h^{\text {min},\Delta R}$ which follows from requiring that the first Higgs arises from the *b*-tagged jets with the smallest $\Delta R$ separation. We require that both $M_h^{\text {min},M},M_h^{\text {min},\Delta R}>30~\text {GeV}$. Finally we only use the Higgs bosons reconstructed upon utilising the minimum mass difference procedure.

Again we input a number of kinematic distributions to the BDT, detailed in Table 7, the results are shown in Table 8. The signal vs. background ratio is extremely small, $O(10^{-3})$, leaving the analysis highly sensitive to systematic uncertainties with only little improvement possible using jet-substructure approaches.

### The boosted channel

We follow here the philosophy of Sect. 2.4, by exploiting the fact that most of the sensitivity to the Higgs self-coupling comes from configurations where the di-Higgs system has a small invariant mass. This can be achieved by requiring the di-Higgs system to recoil against one or more high $p_T^{j}$ jets. If the Higgses have enough transverse momentum, their decay products, the $b \bar{b}$ pairs, will be collimated and eventually will be clustered as large radius jets. Such jets can be identified and disentangled from QCD jets with the use of standard substructure techniques.

Events are first pre-selected by requiring at least two central fat jets with parameter $R=0.8$ that contain at least two b-subjets. The fat jets are selected if $p_T^{j} > 300$ GeV and $|\eta ^{j}| < 2.5$. We assume, as previously, a conservative $70\%$ *b*-tagging efficiency. We further ask the di-fatjet pair to be sufficiently boosted, $p_T^{jj} > 250$ GeV, and the leading jet to have a $p_T^{j_1} > 400$ GeV. Finally, we require that $\Delta R(j_1,j_2) < 3.0$ as well as $(p_T^{j_1} - p_T^{j_2})/p_T^{jj} < 0.9$.

**Fig. 6:**
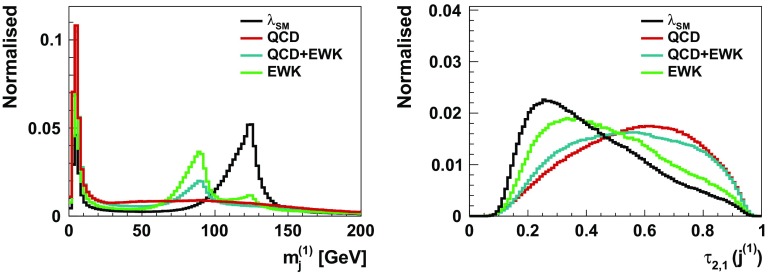
Normalised differential soft-dropped mass $m_{SD}$ (left) and N-subjettiness ratio $\tau _{2,1}$ (right) for the leading reconstructed fat jet. The observables are used for the Higgs-jet tagging

The last steps of the event selection make use of jet-substructure observables and are designed to identify the collimated Higgs fat jets with high purity. The main background contribution is QCD $g \rightarrow b \bar{b}$ events, where configurations are dominated by soft and collinear splittings. The resulting jets are hence often characterized by one hard prong, as opposed to fat jets containing the Higgs decay products, that will feature a clear two-prong structure. The “2” versus “1” prong hypotheses of a jet can be tested with the $\tau _{2,1}$ observable [76]. Moreover Higgs jets typically have an invariant mass close to $m_H = 125$ GeV, as opposed to QCD jets that tend to have a small mass. QCD jets can therefore be rejected by requiring a soft-dropped mass $m_{SD}$ [77] of the order of the Higgs mass. These two observables are shown in Fig. 6 for the leading reconstructed fat-jet. The Higgs-jet tag consists in selecting jets with $\tau _{2,1} < 0.35$ and $100< m_{SD} < 130$ GeV. This simple selection yields a tagging efficiency of 6% and a mistag rate of 0.1%. We apply the Higgs-jet tagging procedure to the two fat jets.

**Table 5:** Results of the boosted $pp\rightarrow hhj$ decay channels outlined in Sect. 2.4 in femtobarns (numbers to the left of the double vertical lines) after an optimised cut on the BDT output. We include results for three different choices of the self-coupling within the $\kappa$ framework [64], $\kappa _\lambda =\lambda /\lambda _{\text {SM}}$; BDT training is performed with $\lambda =\lambda _{\text {SM}}$.

	Signal	QCD+EW	EW	$t\bar{t} j$	Tot. background	*S* / *B*	$S/\sqrt{B}$, 30/ab
$\kappa _\lambda =0.5$	0.428	0.95	0.27	2.31	3.53	0.121	39.44
$\kappa _\lambda =1$	0.363					0.103	33.44
$\kappa _\lambda =2$	0.264					0.075	24.31

**Table 6:** Comparison of an optimised cut-and-count analysis with our BDT analysis of Sect. 2.4. We optimise the selection to obtain a comparable signal yield after all analysis cuts. The order of the selection criteria reflects their relative impact on the BDT score

Cut	Cross section after cut [fb]					
	$\kappa _\lambda =1$	$\kappa _\lambda =0.5$	$\kappa _\lambda =2$	QCD+EW	EW	$t\bar{t} j$
Preselection	0.86	1.09	0.56	11.73	2.20	4090.29
$m_{T2} > 120$ GeV	0.65	0.78	0.45	4.65	1.10	300.68
	0.62	0.74	0.43	4.43	1.05	196.36
$100~{\text {GeV}}< M_{\tau ,\tau } < 150~{\text {GeV}}$	0.48	0.57	0.33	0.96	0.26	28.05
	0.47	0.56	0.32	0.92	0.25	21.75
$\Delta R(b_1 \tau _{\text {vis,1}}) > 0.8$	0.47	0.56	0.32	0.92	0.25	20.28
$p_{T}(H_{\tau _{\text {vis,1}}\tau _{\text {vis,2}}}) > 60.0$ GeV	0.45	0.53	0.31	0.88	0.24	19.02
$\Delta R(b_1 \tau _{\text {vis,2}}) > 0.8$	0.44	0.52	0.31	0.87	0.24	18.91
$100~{\text {GeV}}< M_{\text {inv},\text {filt}} < 150~{\text {GeV}}$	0.32	0.38	0.22	0.43	0.06	5.78
$\Delta R(b_1b_2) > 0.8$	0.32	0.38	0.22	0.43	0.06	5.46
$\Delta R(\tau _{\text {vis,1}} \tau _{\text {vis,2}}) > 0.8$	0.31	0.37	0.22	0.42	0.06	5.15
	0.36	0.44	0.26	0.95	0.27	2.31
	BDT performance [fb]					

The final results for the boosted analysis are summarized in Table 9. Although we find only a mild improvement on the significance compared to the resolved analysis, there is a clear improvement on the signal over background ratio $\sim 0.02$, allowing to better control background systematics.

## Summary and conclusions

Di-Higgs searches and their associated interpretation in terms of new, non-resonant physics are a key motivation for a future high-energy *pp* collider. Recent analyses have mainly focused on direct $pp\rightarrow hh$ production, which has the shortcoming of back-to-back Higgs production generically accessing a phase space region with only limited sensitivity to the modifications of the trilinear Higgs coupling. This situation can be improved by accessing kinematical configurations where a collinear Higgs pair recoils against a hard jet, thus accessing small invariant masses $M_{hh}\simeq 2m_t$ over a broad range of final state kinematics. This is the region where modifications of the trilinear Higgs coupling are most pronounced.

In this work, we have focussed on this *hhj* final state at a 100 TeV collider. As exclusive final state cross sections are small, we focus in particular on the dominant $hh\rightarrow b\bar{b} b\bar{b}$ and $hh\rightarrow b\bar{b} \tau ^+ \tau ^-$ decay channels. Multi-Higgs final states suffer from small rates even in these dominant Higgs decay modes, which necessitates considering multivariate analysis techniques. We find that although the four *b* final state is challenged by backgrounds with some opportunities to enhance sensitivity at large momenta, the $hh\rightarrow b\bar{b} \tau \tau$ final states provide a promising avenue to add significant sensitivity to the search for non-standard Higgs interactions. In particular, the hadronic tau decay channels which can be isolated with cutting-edge reconstruction techniques introduced by the CMS collaboration, drives the sensitivity. Relying on boosted final states, we show that *hhj* production could in principle allow to constrain the Higgs self-coupling at the 8% level at 30/ab (assuming no systematic uncertainties and other couplings to be SM-like). This precision is thus worse than the $\sim 4\%$ result obtained for the inclusive $hh(\rightarrow b\bar{b}\gamma \gamma )$ channel shown in Ref. [34]. Given the complexities of these analyses involving the Higgs self-coupling, we find it important that there be several independent modes to probe its value with a precision below the 10% threshold. Furthermore, the different kinematical regimes probed by the *hh* and the *hhj* measurements could be sensitive in different ways to possible deviations from the SM expectations. This motivates $pp\rightarrow hh j$ with semi-leptonic tau decays as an additional main search channel for modified Higgs physics.

**Table 7:** Observables included to the boosted decision tree for the fully-resolved 4 *b*-jet analysis of $pp\rightarrow hhj$, Sect. 3

Observable	Reconstructed object
$p_T$	4 *b*-tagged jets
	Hardest non *b*-tagged jet
	Reconstructed $h\rightarrow b\bar{b}$ for both $M_h^{\text {min},M}$ definition
$p_T$ ratio	4 *b*-tagged jets taken in pairs
$\Delta R$	*b*-tagged jets
	*b*-tagged jets and non-*b*-tagged jet
*M*	4 *b*-tagged jets
$M_h^{\text {min},M}$	See text
$\Delta \phi$	Between $h\rightarrow b\bar{b}$ for the $M_h^{\text {min},M}$ definition

**Table 8:** Results for the fully-resolved 4 *b*-jet analysis of $pp\rightarrow hhj$, Sect. 3. We include results for three different choices of the self-coupling within the $\kappa$ framework [64], $\kappa _\lambda =\lambda /\lambda _{\text {SM}}$; BDT training is performed with $\lambda =\lambda _{\text {SM}}$. Numbers to the left of the double vertical lines are in femtobarns

	Signal	QCD	QCD+EW	EW	Tot. background	$S/B\times 10^3$	$S/\sqrt{B}$, 30/ab
$\kappa _\lambda =0.5$	0.252	41.67	1.86	0.13	43.66	5.8	6.61
$\kappa _\lambda =1$	0.230					5.3	6.03
$\kappa _\lambda =2$	0.160					3.6	4.18

**Table 9:** Results for the boosted 4 *b*-jet analysis of $pp\rightarrow hhj$, Sect. 3. We include results for three different choices of the self-coupling within the $\kappa$ framework [64], $\kappa _\lambda =\lambda /\lambda _{\text {SM}}$. Numbers to the left of the double vertical lines are in femtobarns

	Signal	QCD	QCD+EW	EW	Tot. background	$S/B\times 10^3$	$S/\sqrt{B}$, 30/ab
$\kappa _\lambda =0.5$	0.094	4.3	0.1	0.003	4.4	20.8	7.67
$\kappa _\lambda =1$	0.085					19.1	6.61
$\kappa _\lambda =2$	0.071					16.2	5.85

## Footnotes

1 We impose $p_T(j) > 100$ GeV at the parton level.

2 The top decays to $b, \ell \; \text {and} \; \nu$ have been implemented as decay chains in the MadGraph5_aMC@NLO framework, at the production level.

3 The “EW” and “QCD+EW” processes correspond in MadGraph5_aMC@NLO to the interaction orders QED $=$ 4 QCD $=$ 1 (pure EW) and QED $=$ 2 QCD $=$ 3 (mixed QCD+EW) respectively. We note that both classes of processes include single-Higgs production contributions.

4 Jets are defined through the anti-*kT* algorithm [59, 60] with a jet resolution parameter 0.4 inside the rapidity range $|\eta ^j| < 4.5$. For the *b*-tagging efficiency we choose 60% at a 2% mistagging rate, which is a realistic at the LHC [61]. Isolated muons and electrons are defined by requiring a small hadronic energy deposit in the vicinity of the lepton candidate, $E^{\text {had}}/p_T^{\ell }<10\%$ within $\Delta R<0.2$.

5 The redundant variables which do not affect the significance are: $p_T^{b_2}, p_T^{\ell _2}, \Delta R (b_2 \ell _2), \Delta R (\ell _2 j)$ and $\Delta \phi (bb,\ell \ell )$, where the index refers to the $p_T$ ordering of an object.

6 We have checked our results for overtraining.

7 The redundant variables for this case are: $\Delta R (b_2 \ell ), \Delta R (b_2 \tau _h), \Delta R (b_1 j), \Delta R (b_2 j)$ and $\Delta R (\tau _h j)$.

8 Furthermore, we also require light jets faking *b*-jets to have $p_T > 25$ GeV.

9 Here also we obtain no change in sensitivity upon removing the redundant variables, *viz.*, $p_{T,2}^{\text {filt}}, p_T^{\tau _{\text {vis},1}}, p_T^j, \Delta R (b_2 j), \Delta R (\tau _{\text {vis},1} j), \Delta R (b_1 j)$ and $\frac{p_T^{\tau _{\text {vis},2}}}{p_T^{\tau _{\text {vis},1}}}$.

10 For instance, the recently reported evidence of $t\bar{t} h$ production [74] crucially relies on neural net analyses.

11 Jets are defined as in Sect. 2.2.
